# Proteomics analysis reveals the differential protein expression of female and male adult *Toxocara canis* using Orbitrap Astral analyzer

**DOI:** 10.1186/s40249-024-01246-9

**Published:** 2024-10-09

**Authors:** Hui-Jie Qiu, Ya-Jia Zhou, Zhi-Yu Li, Yi-Han Lv, Xing-Quan Zhu, Wen-Bin Zheng

**Affiliations:** Laboratory of Parasitic Diseases, College of Veterinary Medicine, Shanxi Agricultural University, Taigu, Shanxi Province 030801 People’s Republic of China

**Keywords:** *Toxocara canis*, Toxocariasis, Zoonosis, Orbitrap Astral, Proteomics, Reproduction

## Abstract

**Background:**

*Toxocara canis*, the most prevalent helminth in dogs and other canines, is one of the socioeconomically important zoonotic parasites, particularly affecting pediatric and adolescent populations in impoverished communities. However, limited information is available regarding the proteomes of female and male adult *T. canis*. To address this knowledge gap, we performed a comprehensive proteomic analysis to identify the proteins with differential abundance (PDAs) and gender-specifically expressed proteins between the two sexes adult *T. canis*.

**Methods:**

The comparative proteomic analysis was carried out by the Orbitrap mass spectrometry (MS) with asymmetric track lossless (Astral) analyzer. The difference analysis was conducted using *t*-test and the proteins verification was achieved through parallel reaction monitoring (PRM). The potential biological functions of identified adult *T. canis* proteins and PDAs were predicted by Gene Ontology (GO) and Kyoto Encyclopedia of Genes and Genomes (KEGG) databases. The domain, transcription factor and subcellular localization of the identified proteins and PDAs were analyzed by InterPro, AnimalTFDB 4.0 and Cell-mPLOC 2.0 databases, respectively.

**Results:**

A total of 8565 somatic proteins of adult *T. canis* were identified. Compared to male adult, 682 up-regulated PDAs and 844 down-regulated PDAs were identified in female adult with *P*-values < 0.05 and |log_2_^FC^| > 1, including 139 proteins exclusively expressed in female and 272 proteins exclusively expressed in male. The GO annotation analysis using all PDAs revealed that the main biological processes, cellular components and molecular functions corresponded to aminoglycan metabolic process, extracellular region and protein tyrosine phosphatase activity, respectively. The KEGG analysis using all PDAs showed that the pathways were mainly associated with adipocytokine signaling pathway, proximal tubule bicarbonate reclamation and PPAR signaling pathway.

**Conclusions:**

This study reveals the differential protein expression between female and male adult *T. canis*, providing valuable resource for developing the novel intervention strategies against *T. canis* infection in humans and animals, especially from the perspective of sexual development and reproduction.

**Graphical abstract:**

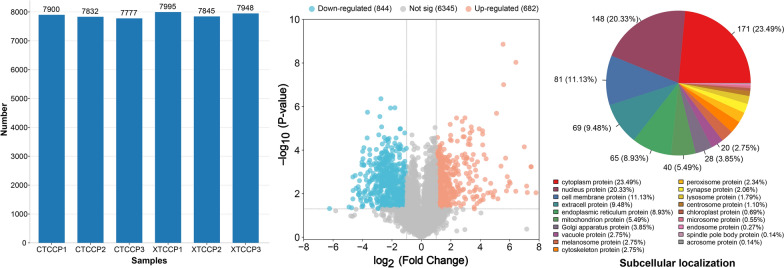

**Supplementary Information:**

The online version contains supplementary material available at 10.1186/s40249-024-01246-9.

## Background

*Toxocara canis*, one of the most neglected parasitic zoonoses, is wildly distributed all over the world with a significant socioeconomic impact, especially in impoverished communities [1]. This parasite can infect humans, causing ocular larva migrans, visceral larva migrans and neurotoxocariasis, especially in pediatric and adolescent populations [2]. Globally, the prevalence of *Toxocara* infection is ~19% in humans, with higher rates observed in children [3]. Dogs and other canines are the definitive hosts of *T. canis*, where adult *T. canis* mainly inhabit the small intestines, surviving for several months to years [4]. The global prevalence of *T. canis* infection is reported to be 11.1% in dogs [5]. The adult female *T. canis* can shed thousands of eggs per day through the faeces, contaminating the public environment, food, water or vegetables [6]. Under suitable temperature and humidity conditions, these eggs can develop into embryonated eggs that can infect humans and other paratenic/definitive hosts [7]. Previous pooled incidence revealed that 7% vegetables, 10% cucurbits and 21% soil samples of public environment are polluted worldwide by *Toxocara* eggs [8, 9], posing a significant threat to human and animal health.

Advancements in genomics and proteomics provide unprecedented opportunities to uncover biological phenomena and promote human and animal health. Our previous study sequenced and annotated the draft genome of *T. canis*, and identified the stage transcription profiles (adult and third-stage larvae) and gender-enriched transcription profiles of *T. canis* by next generation sequencing (NGS) [10], which provides basic data for further exploring the molecular biology in *T. canis* development and the host-parasite interaction. In addition, the differential genes between the female and male adult *T. canis* were further identified by another independent study [11], which provides a useful basis to explore the reproductive biology of *T. canis*. Subsequently, a wide range of somatic proteins of *T. canis* larvae were identified by liquid chromatography-mass spectrometry/mass spectrometry (LC-MS/MS) [12], which facilitates the research on larval development, metabolism and survival within the host. However, little is known of the differential protein expression between female and male *T. canis*. With the continuous development of “Omics” technologies, proteomics technologies, such as isobaric tags for relative and absolute quantification (iTRAQ), tandem mass tag (TMT), label free and data-independent acquisition (DIA), have been wildly applied to study the various biological phenomena, such as analyzing the difference of casein fraction in human milk associated with infant gender [13], comparing the difference between fresh and frozen thawed spermatozoa [14], and spatially mapping the proteomes of *Trypanosoma brucei* and *T. congolense* [15]. The innovation of “Omics” technologies will undoubtedly promote the progress of molecular biology. A new narrow-window DIA (nDIA) strategy was established in 2023, which consists of high-resolution MS1 scans with parallel tandem MS (MS/MS) scans of ~200 Hz using 2-Th isolation windows, implementing the DIA analysis by using data-dependent acquisition (DDA) technique [16]. The nDIA strategy is achieved by pairing a quadrupole Orbitrap mass spectrometer with the asymmetric track lossless (Astral) analyzer (Orbitrap Astral) which provides faster throughput, deeper coverage, higher sensitivity, and low-ppm mass accuracy for quantitative proteomics studies [16, 17].

In the present study, we compared the protein expression profiles between the two sexes of adult *T. canis* by Orbitrap Astral platform to identify the proteins with differential abundance (PDAs) and gender-specifically expressed proteins between the female and male adult *T. canis*. Through comprehensive proteomics analysis, we characterized the proteome, elucidated the functions of PDAs, and identified the key molecular pathways. These results provide the critical resources for better understanding of *T. canis* pathobiology, facilitating the development of novel intervention strategies for toxocariasis, especially from the perspective of sexual development and reproduction.

## Methods

### Sampling and materials

To collect adult *T. canis*, a 2-month-old male Beagle dog was used. The dog was experimentally infected with 2000 infectious eggs of *T. canis*. At 90 days post-infection (dpi), the dog was anesthetized by Zoletil 50 (Virbac, Nice, Franch) with a dosage of 15 mg/kg. Subsequently, the dog was humanely euthanized by injecting 10% KCl with a dosage of 0.75 mg/kg under general anesthesia as previously described [18]. Adult *T. canis* were recovered from the dog's small intestine and washed three times with PBS. The female and male adult *T. canis* were identified and separated by observing the distribution and location of the uterus or testis, and subsequently divided into female group and male group. Each gender group comprised three biological replicates for subsequent protein extractions. To remove host contaminants, the female and male *T. canis* were cultured in PBS at 37 °C for 1 h. Subsequently, each intact and vibrant adult *T. canis* was individually placed in a freezer tube, frozen in liquid nitrogen, and stored at − 80 °C.

### Extraction and trypsin treatment of adult T. *canis* proteins

Each intact adult *T. canis* was grounded individually in liquid nitrogen and lysed with SDT (containing 100 mmol/l NaCl) and 1/100 volume of DTT, followed by 5 min of ultrasonication on ice for subsequent protein extractions. The lysate was incubated for 10 min at 95 °C, and then placed on ice for 2 min. It was subsequently centrifuged at 12,000 × *g* for 15 min at 4 °C. The supernatant of lysate was alkylated with IAM for 1 h at 24 °C in dark room. Then, the sample was completely mixed with four times volume of precooled acetone and incubated at − 20 °C for 2 h. After centrifugation at 12,000 × *g* for 15 min at 4 °C, the pellet was collected, washed with 1 ml cold acetone, and dissolved completely in dissolution buffer. The concentration and quality of extracted *T. canis* proteins were assessed using a Bradford assay and 12% SDS-PAGE gel electrophoresis, respectively. For digestion, 50 μg *T. canis* proteins were diluted to 100 μl by dissolution buffer containing 8 mol/L Urea and 100 mmol/L TEAB (pH 8.5). The protein mixture was digested with 1 μg trypsin and 100 mmol/l TEAB buffer at 37 °C for 4 h, followed by an overnight digestion with 1 μg trypsin and 0.1% CaCl_2_. The digested proteins were acidified with formic acid (FA) to adjust pH below 3, followed by centrifuged at 12,000 × *g* for 5 min at 24 °C, and the supernatant was slowly loaded to the C18 desalting column [19]. The column was washed three times with a washing buffer [0.1% FA, 3% acetonitrile (ACN)], and the digested proteins were collected with an elution buffer (0.1% FA, 70% ACN), then lyophilized.

### Vanquish neo UHPLC-astral LC/MS DIA method

The lyophilized protein powder from each *T. canis* was re-dissolved in 10 µl of mobile phase A (0.1% FA) and centrifuged at 14,000 × *g* for 20 min at 4 °C. A 200 ng aliquot of the supernatant was injected into a Vanquish Neo upgraded UHPLC system equipped with a C18 pre-column of 174,500 (5 mm × 300 μm, 5 μm, Thermo Fisher Scientific, Waltham, USA) maintained at 50 °C in a column oven. The separation was performed using a C18 analytical column of ES906 (PepMap TM Neo UHPLC 150 µm × 15 cm, 2 μm, Thermo Fisher Scientific). Mobile phase B consisted of 0.1% FA and 80% ACN. The separation was achieved using the following gradient elution program: 96% mobile phase A at 2.5 µl/min for 0–0.2 min, 96% mobile phase A at 1.3 µl/min for 0.2–0.3 min, 92% mobile phase A at 0.8 µl/min for 0.3–14.2 min, 77.5% mobile phase A at 0.8 µl/min for 14.2–21.1 min, 65% mobile phase A at 0.8 µl/min for 21.1–21.5 min, 45% mobile phase A at 2.5 µl/min for 21.5–21.9 min, 1% mobile phase A at 2.5 µl/min for 21.9–22.6 min.

The liquid phase separation end was directly connected to an Orbitrap Astral platform for mass spectrometric detection (Thermo Fisher Scientific). The separated peptides proceed directly into the mass spectrometer for DIA analysis after ionizing by Easy-spray ion (ESI) source [16]. The ion spray voltage is set to 2.0 kV, and the ion transfer tube temperature is maintained at 290 °C. The mass spectrum was in a data-independent acquisition (DIA) mode, the main settings are as follows: the full first-stage MS scan range is 380–980 m/z, the primary MS resolution is set to 240,000 (200 m/z), the automatic gain control (AGC) is set to 500%, and the parent ion selection window is set to 2-Th, the number of DIA windows is set to 300, the normalized collision energy was set to 25%; and the secondary MS scan range is 150–2000 m/z, the sub-ion resolution Astral is set to 80,000, and the maximal injection time is 3 ms.

### Identification and quantitation of T. *canis* proteins

The MS/MS data were searched according to the *T. canis* protein database, downloaded from UniProtKB (released on March 6, 2024), using DIA-NN software 1.8 (Cambridge Centre for Proteomics, Cambridge, UK) with a mass tolerance of 10 mg/L for precursor ions and 0.02 Da for fragment ions [20]. Cysteine alkylation was designated as a fixed modification, while methionine oxidation and N-terminal acetylation, loss of methionine and loss of methionine + acetylation modification were set as variable modifications. The search results were further filtered by DIA-NN software, retaining only credible Peptide Spectrum Matches (PSMs) with a confidence level of 99% or higher to ensure the quality of the analytical results. False discovery rate (FDR) validation was performed to exclude peptides and proteins with an FDR greater than 1%. Retention time correction was performed using indexed Retention Time (iRT) standards to enable consistent protein quantification across different samples. Proteome data was analyzed by principal component analysis (PCA) and coefficient of variance (CV) analysis. To ensure data quality, proteins detected in only one out of the three biological replicates within the same group were removed. The proteins with differential abundance (PDAs) were identified using the following criteria: *P*-values < 0.05 (*t*-test) and |log_2_^FC^| > 1.

### Verification of T. *canis* proteins identified through nDIA analysis using PRM

To validate the results obtained from the nDIA-based proteomic analysis, twenty PDAs and ten proteins showing no differential abundance were selected for parallel reaction monitoring (PRM) verification. Initially, an equal amount of digested peptide from each sample was mix. Mobile phases A (pH adjusted to 10.0 using NH_3_·H_2_O) and B (100% ACN) were used to develop an elution buffer. The tryptic peptides were sequentially separated using a homemade Durashell RP column with 2 mg packing (3 µm, 150 Å, Agela Technologies, Tianjin, China) in a 200 µl tip, utilizing elution gradients ranging from 3 to 35% mobile phase B. Subsequently, the eluted peptides were combined into three fractions and dried under vacuum.

Each fraction (1 µg) was initially analyzed using a Label-free method on an EASY-nLC™ 1200 UHPLC system (Thermo Fisher Scientific) coupled with a Q Exactive™ HF-X mass spectrometer (Thermo Fisher Scientific) for pre-experiment. The top 40 most abundant precursor ions were selected in full scan mode for fragmentation by higher energy collisional dissociation (HCD), followed by MS/MS analysis at a resolution of 15,000 (200 m/z) with AGC target value of 5 × 10^4^, maximum ion injection time of 45 ms, normalized collision energy of 27%, intensity threshold of 2.2 × 10^4^, and dynamic exclusion set to 20 s. The off-line data was searched using Proteome Discoverer (PD) software 2.5 (Thermo Fisher Scientific) with missed cleavages set to zero, and one to three unique peptides were selected for each protein. Following peptide selection, the mixed peptides were re-detected using the same chromatographic method and instrument as above Label-free. The mass spectrometry method included one full scan followed by one PRM scan using the Q Exactive™ HF-X mass spectrometer. Information for the target peptide (e.g. m/z, charge number and charge type) were input into the inclusion list. Full scan was set as resolution of 60,000 (200 m/z) with AGC target value of 3 × 10^6^ and maximum ion injection time of 20 ms. The PRM was set as resolution of 15,000 (200 m/z) with AGC target value of 1 × 10^5^, maximum ion injection time of 100 ms, normalized collision energy of 27%. The off-line data was analyzed by Skyline software 22.2 (University of Washington, Seattle, USA) to assess the reproducibility and stability of selected peptides.

After confirmation of experimental details, an equal amount of trypsin-treated peptides from each sample was spiked with a labeled peptide DSPSAPVNVT**V**R (bold V for heavy isotope labeling) as an internal standard. The formal PRM analysis experiment commenced following the UHPLC-MS/MS protocol outlined above, with samples analyzed using one full scan followed by one PRM scan pattern. The off-line data was analyzed by Skyline software, and the peak area was corrected using the internal standard peptide.

### Bioinformatics analysis of adult *T. canis* proteins

The potential biological functions and protein domain of identified adult *T. canis* proteins and PDAs were predicted and analyzed using GO (http://www.geneontology.org/), KEGG (https://www.kegg.jp/) and InterPro (IPR, https://www.ebi.ac.uk/interpro/) database, with InterProscan software 5.22–61.0 (European Bioinformatics Institute, Hinxton, UK) [21–24]. The transcription factor (TF) and the subcellular localization were identified using the AnimalTFDB 4.0 (http://bioinfo.life.hust.edu.cn/AnimalTFDB4/) and Cell-mPLOC 2.0 (http://www.csbio.sjtu.edu.cn/bioinf/Cell-PLoc-2/) databases, respectively [25, 26].

## Results

### Quantification and validation of adult *T. canis* proteins

**Fig. 1:**
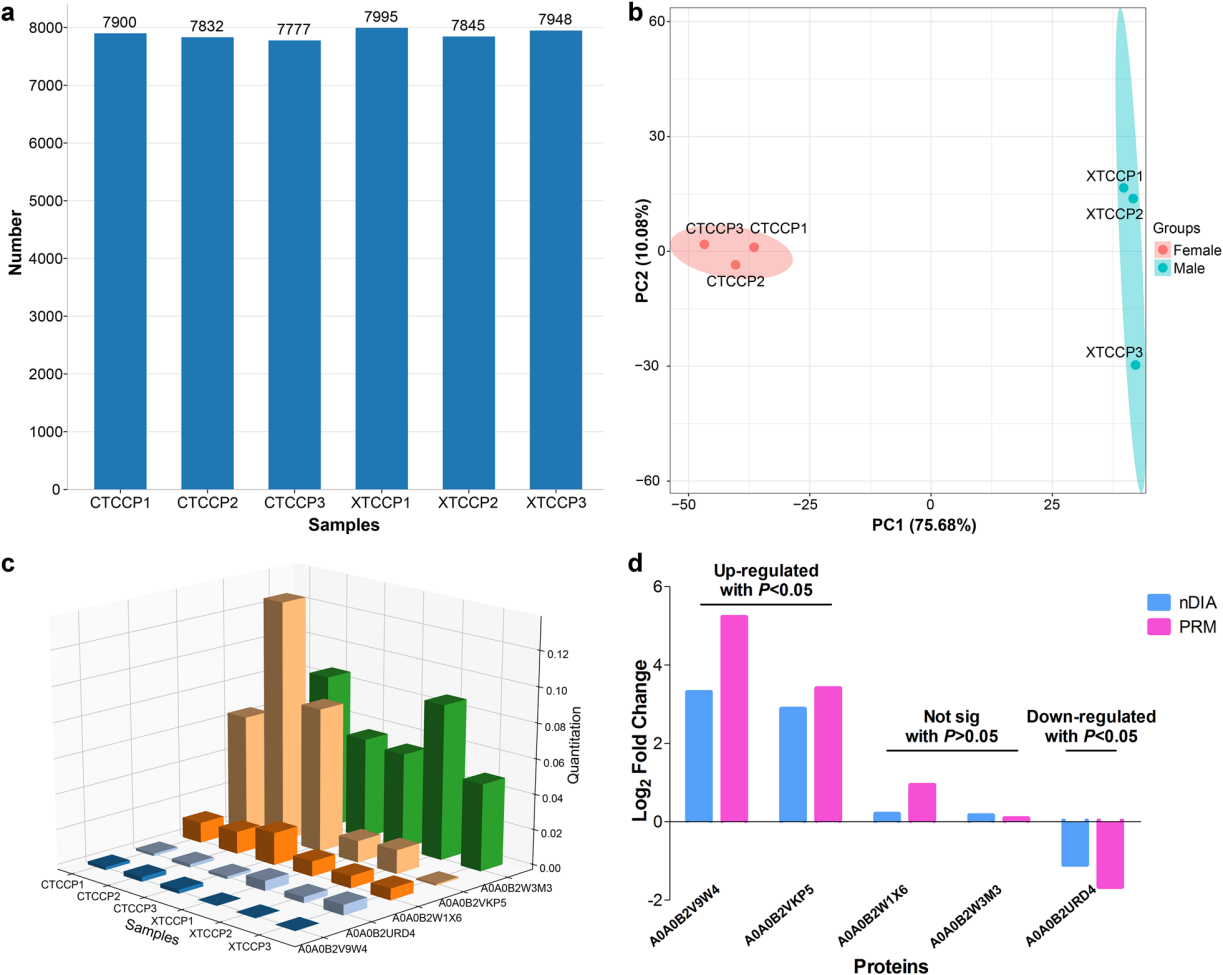
The quantification and validation of adult *Toxocara canis* proteins. **a** The number of *T. canis* proteins identified from each sample using narrow-window data-independent acquisition (nDIA). **b** The principal component analysis (PCA) of proteome data among the six samples. **c** The quantitation of five *T. canis* proteins in each sample using parallel reaction monitoring (PRM). **d** The verification of *T. canis* proteins identified through nDIA analysis using PRM

A total of 82,238 peptides and 8565 *T. canis* proteins were identified (Additional file 1: Table S1). The number of *T. canis* proteins detected in each sample remained relatively stable, ranging from 7777 to 7995 proteins (Fig. 1a). The PCA revealed a distinct separation between female and male adult *T. canis* (Fig. 1b). The CV analysis showed that the trend of CV cumulative curve rises faster, indicating good overall repeatability of the sample (Additional file 2: Fig. S1). After conducting a pre-experiment LC–MS/MS analysis for PRM verification, considering peptide uniqueness, five proteins were identified as suitable for the formal PRM verification experiment (Fig. 1c). The expression trends of these proteins in PRM were consistent with those obtained in nDIA, supporting the validity of our proteomics data (Fig. 1d).

### Functional prediction of adult *T. canis* proteins

**Fig. 2:**
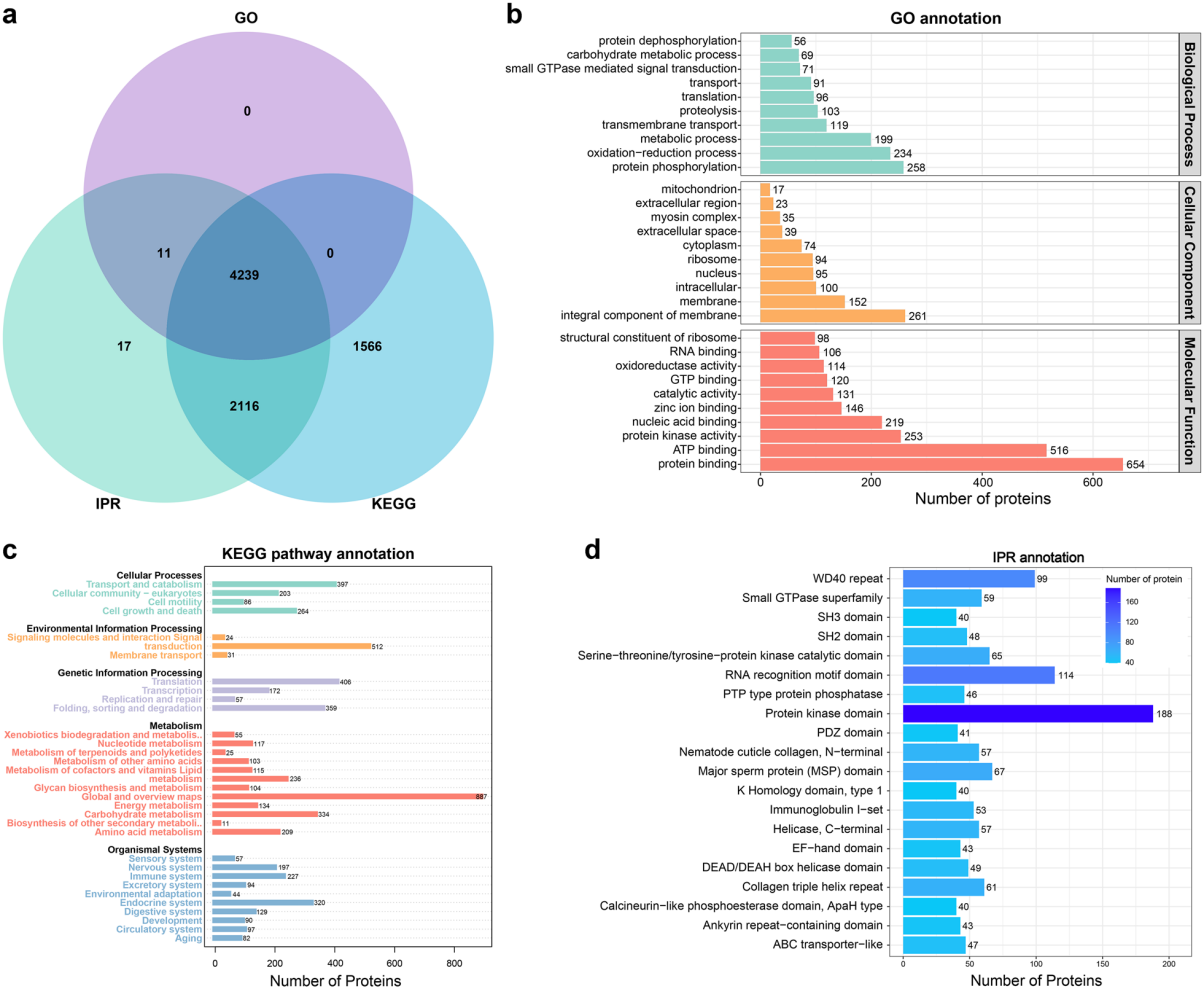
The functional prediction of adult *Toxocara canis* proteins. **a** The Venn diagrams showing the common and exclusive proteins annotated by GO, KEGG and IPR database. The top 30 enriched GO terms (including BP, CC and MF categories) (**b**), all enriched KEGG terms of level 2 signaling pathways (**c**) and top 20 protein domain (**d**) of all identified adult *T. canis* proteins

The functional annotation of the 8565 adult *T. canis* proteins identified in this study was performed using the GO, KEGG and IPR databases. Of these, 7949 proteins were successfully annotated (Fig. 2a). Among them, 4250 proteins were enriched in 928 GO items by GO annotation, including 2292 proteins in 364 “biological process” items, 1110 proteins in 136 “cellular component” items, and 3471 proteins in 428 “molecular function” items (Additional file 1: Table S2), and the top 10 GO terms of each classification are shown in Fig. 2b. 7921 proteins were annotated by KEGG database, and 2682 proteins were enriched in 33 level 2 signaling pathways by KEGG pathway analysis. Among these, “global and overview maps” was highly enriched with 887 proteins in the item of “metabolism”; “signal transduction” was highly enriched with 512 proteins in the item of “environmental information processing”; “translation” was highly enriched with 406 proteins in the item of “genetic information processing”; “transport and catabolism” was highly enriched with 397 proteins in the item of “cellular processes”; “endocrine system” was highly enriched with 320 proteins in the item of “organismal systems”, and no any protein was enriched in the item of “human diseases” (Additional file 1: Table S3), and all the KEGG terms of level 2 signaling pathways are shown in Fig. 2c. Furthermore, 6383 proteins were characterized by 3008 domain terms by IPR functional analysis, including 188 proteins with “protein kinase domain”, 114 proteins with “RNA recognition motif domain”, 99 proteins with “WD40 repeat”, 67 proteins with “major sperm protein (MSP) domain” and 65 proteins with “serine-threonine/tyrosine-protein kinase catalytic domain” (Additional file 1: Table S4), and the top 20 protein domains are shown in Fig. 2d.

**Fig. 3:**
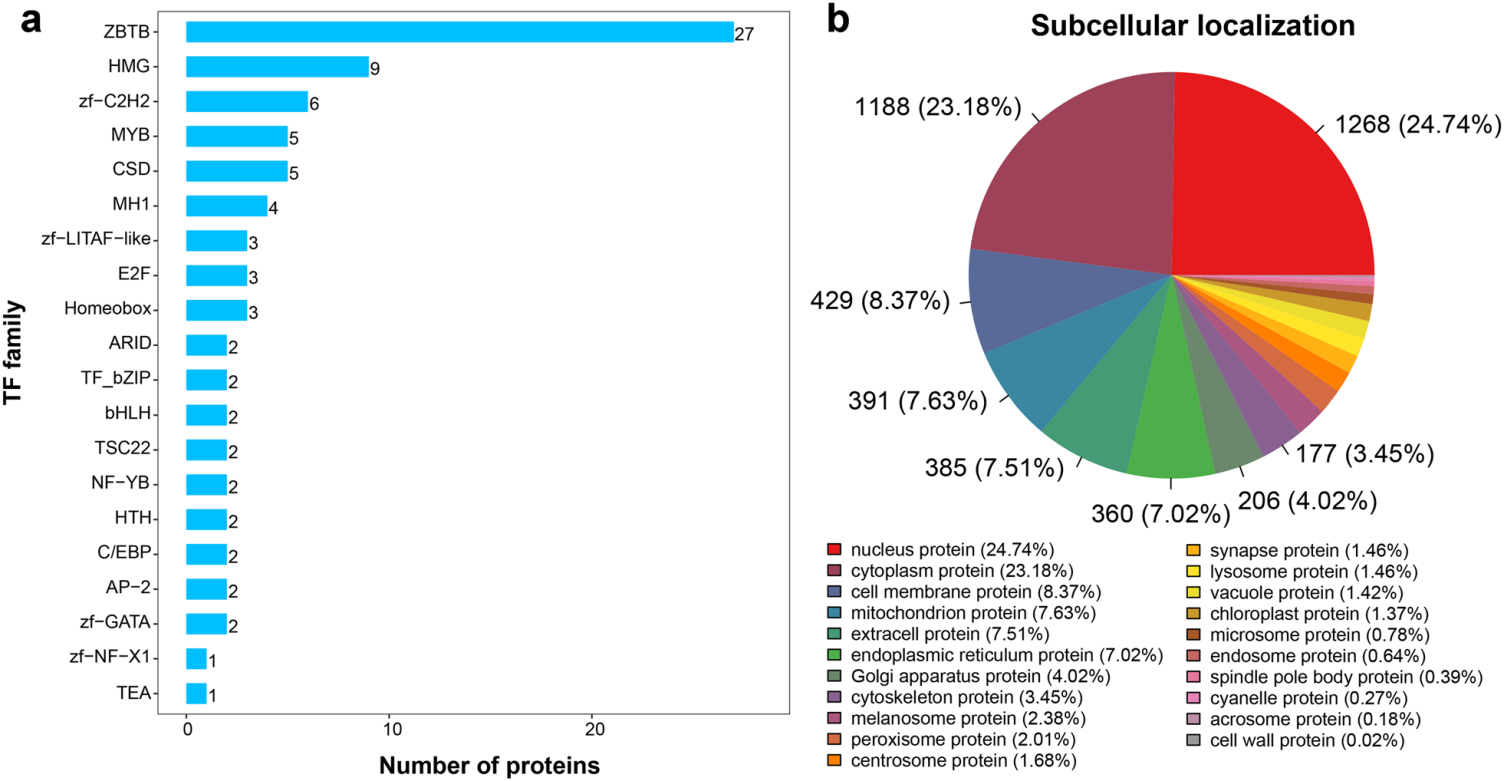
The top 20 transcription factors (TFs) families (**a**) and the subcellular localization (**b**) of all identified adult *Toxocara canis* proteins

A total of 93 transcription factors (TFs) from 28 TF families were identified in this study. Among these, there were 27 ZBTB family TFs, 9 HMG family TFs and 6 zf-C2H2 family TFs (Additional file 1: Table S5), and the top 20 TF families are shown in Fig. 3a. Moreover, the subcellular localization of all identified adult *T. canis* proteins was predicted to explore the potential biological function. A total of 5125 adult *T. canis* proteins were divided into 21 categories, mainly including 1268 “nucleus protein” (24.74%), 1188 “cytoplasm protein” (23.18%), 429 “cell membrane protein” (8.37%), 391 “mitochondrion protein” (7.63%) and 385 “extracell protein” (7.51%) (Fig. 3b and Additional file 1: Table S6).

### Identification of PDAs between female and male adult *T. canis*

**Fig. 4:**
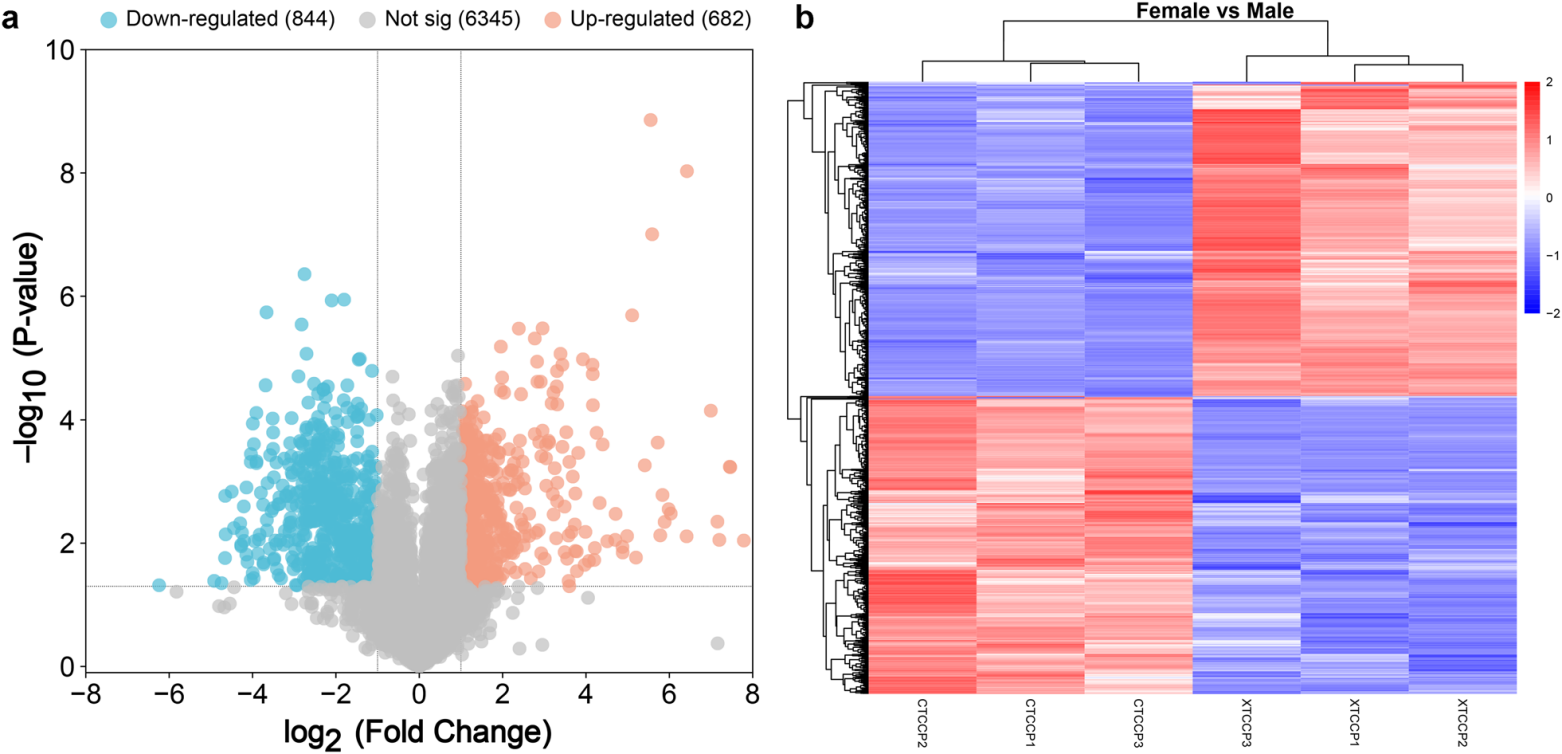
The volcano plots (**a**) and heatmaps (**b**) showing the proteins with differential abundance (PDAs) identified between female and male adult *Toxocara canis*

Following the exclusion of proteins identified in only one of the three biological replicates within the same group, a total of 7871 *T. canis* proteins were identified for subsequent data analyses (Additional file 1: Table S1). Of these, 7599 proteins were identified in female adult *T. canis* and 7732 proteins were identified in male adult *T. canis*. Among these, 7460 proteins were common to both female and male adult *T. canis*, while 139 proteins were exclusively expressed in female *T. canis* and 272 proteins were exclusively expressed in male *T. canis* (Additional file 3: Fig. S2). A comprehensive analysis revealed a total of 1526 PDAs between female and male adult *T. canis*, including 682 up-regulated PDAs and 844 down-regulated PDAs (Fig. 4 and Additional file 1: Table S7).

### GO annotation and KEGG enrichment analysis of PDAs

**Fig. 5:**
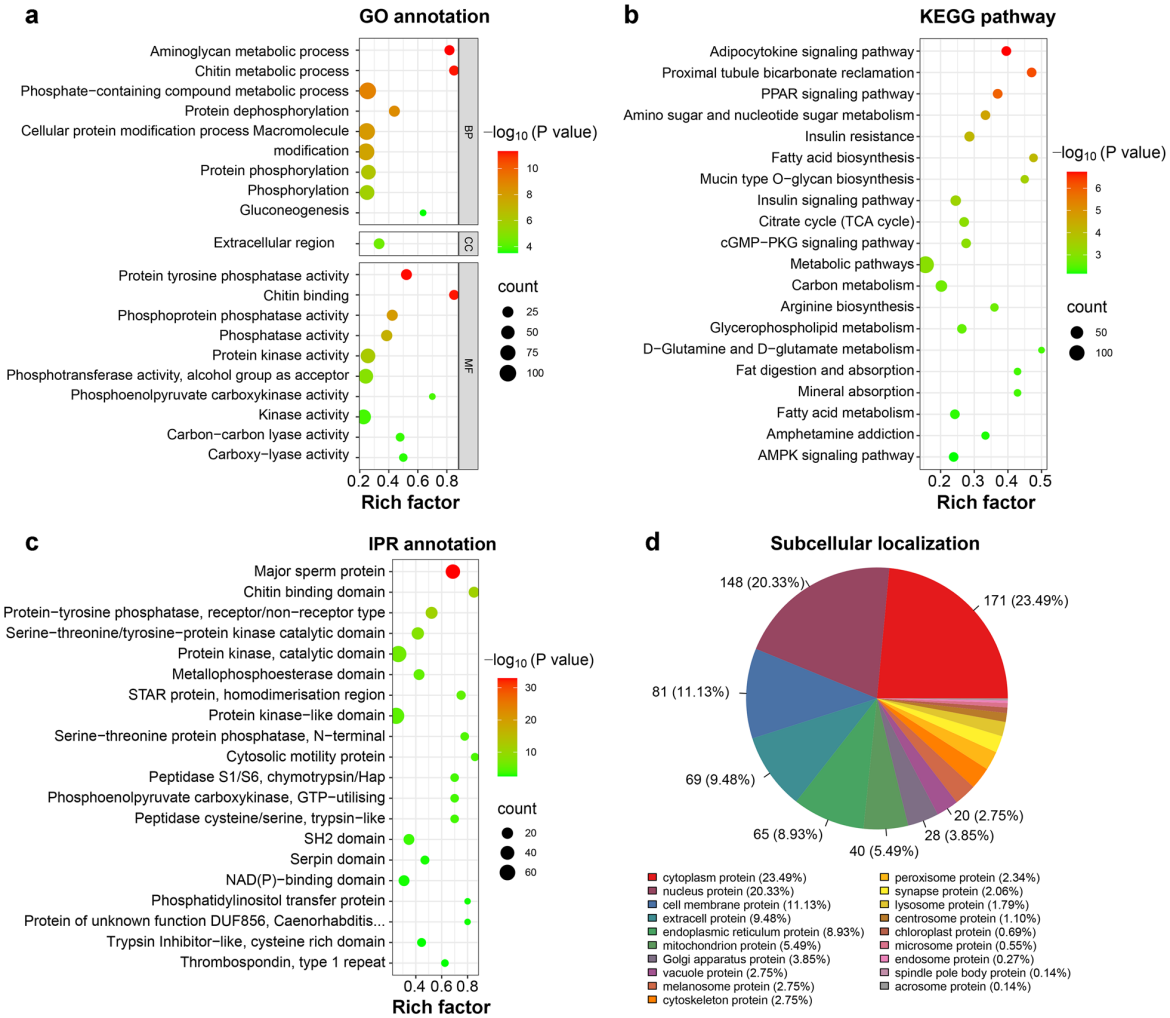
The functional prediction of proteins with differential abundance (PDAs) identified between female and male adult *Toxocara canis*. Scatter plots of the top 20 significantly enriched (*P*-values < 0.05) GO terms (**a**), top 20 significantly enriched (*P*-values < 0.05) KEGG pathways (**b**) and top 20 protein domain (**c**) of PDAs. The X-axis label represents the rich factor; the Y-axis label shows the terms name. The rich factor reflects the proportion of PDAs in each term. The greater the rich factor, the greater the degree of term enrichment. The color of the dots represents the enrichment score [− log_10_^(*P*value)^], where red color indicates high enrichment, while green color indicates low enrichment. Dot size represents the number of PDAs in the corresponding term (bigger dots indicate larger PDAs numbers). **d** The subcellular localization of PDAs

Among the 682 up-regulated PDAs and 844 down-regulated PDAs, 353 PDAs were significantly enriched (*P*-values < 0.05) in 44 GO terms. In the category of biological process, the terms of “aminoglycan metabolic process”, “chitin metabolic process” and “carbohydrate derivative metabolic process” were significantly enriched (*P*-values < 0.05) with many up-regulated PDAs, while the terms of “phosphate-containing compound metabolic process”, “protein dephosphorylation” and “cellular protein modification process” were significantly enriched (*P*-values < 0.05) with many down-regulated PDAs. In the category of cellular component, the terms of “extracellular region”, “mRNA cleavage factor complex” and “proteinaceous extracellular matrix” were significantly enriched (*P*-values < 0.05). In the category of molecular function, the terms of “chitin binding”, “translation release factor activity” and “uridylyltransferase activity” were significantly enriched (*P*-values < 0.05) with many up-regulated PDAs; while the terms of “protein tyrosine phosphatase activity”, “phosphoprotein phosphatase activity” and “phosphatase activity” were significantly enriched (*P*-values < 0.05) with many down-regulated PDAs (Additional file 1: Table S8). The top 20 significantly enriched (*P*-values < 0.05) GO terms are shown in Fig. 5a. Further analysis revealed that among the 1526 PDAs, 201 PDAs with 111 up-regulated PDAs and 90 down-regulated PDAs were significantly enriched (*P*-values < 0.05) in 45 KEGG pathways, including adipocytokine signaling pathway, proximal tubule bicarbonate reclamation, and PPAR signaling pathway (Additional file 1: Table S9). The top 20 significantly enriched (*P*-values < 0.05) KEGG pathways are shown in Fig. 5b.

### The domain, transcription factor and subcellular location analysis of PDAs

Among the 1526 PDAs, 396 PDAs were significantly enriched (*P*-values < 0.05) in 65 domain terms, including “major sperm protein”, “chitin binding domain” and “protein-tyrosine phosphatase, receptor/non-receptor type”. Besides, some PDAs were found to contain multiple domains, such as A0A0B2VUP4 (female germline-specific tumor suppressor gld-1) with 5 domains, and 16 tyrosine-protein kinase with 4 domains (Additional file 1: Table S10). The top 20 significantly enriched (*P*-values < 0.05) domain terms are shown in Fig. 5c. In this study, we identified 21 TFs with differential abundance, belonging to 10 TF families, with 10 up-regulated PDAs and 11 down-regulated PDAs between female and male adult *T. canis*, as well as 3 TFs (e.g. A0A0B2VQ53, A0A183UMK5 and A0A183V3J6) exclusively expressed in female *T. canis* and 3 TFs (e.g. A0A0B2V842, A0A183VAA5 and A0A183V625) exclusively expressed in male *T. canis* (Additional file 1: Table S11). Moreover, the subcellular localization of PDAs was predicted to explore the potential biological function, and a total of 728 PDAs were divided into 19 categories. The predominant categories include “cytoplasm protein” with 171 PDAs (81 up-regulated PDAs and 90 down-regulated PDAs), “nucleus protein” with 148 PDAs (71 up-regulated PDAs and 77 down-regulated PDAs), “cell membrane protein” with 81 PDAs (36 up-regulated PDAs and 45 down-regulated PDAs), “extracell protein” with 69 PDAs (46 up-regulated PDAs and 23 down-regulated PDAs), and “endoplasmic reticulum protein” with 65 PDAs (40 up-regulated PDAs and 25 down-regulated PDAs) (Fig. 5d and Additional file 1: Table S12).

## Discussion

*T. canis* is a zoonotic roundworm found worldwide, capable of causing severe clinical manifestations such as blindness and neurological disorders, especially in pediatric and adolescent populations from impoverished communities [27, 28]. The advent of high throughput sequencing technique, coupled with advanced bioinformatics, has significantly facilitates the research of *T. canis*, an economically significant parasite [29]. In 2015, we sequenced and annotated the draft genome of *T. canis*, revealing at least 18,596 protein coding genes, including 355 TFs and 870 excretory-secretory proteins (ESPs) with various functions [10]. Over the past few decades, mass spectrometry (MS)-based proteomics has become crucial for elucidating the dynamic and diverse proteomic profiles of cells and tissues, despite limitations in capturing the entire proteome. In the last decade, improvements in sample preparation, peptide separations, data processing, and the development of faster and more sensitive MS instruments have pushed the boundaries of depth and throughput in proteomics [30]. Now, one-hour human proteome has reached by using the new Orbitrap Astral platform, ushering unprecedented studies of biological impact by harmony of throughput, depth and sensitivity [31]. In this study, we identified 8565 proteins from adult *T. canis* using the Orbitrap Astral platform, which is a substantial increase compared to the 582 somatic proteins identified from the L3 larvae by the Q Exactive Orbitrap platform in a previous study [12]. Comparing with the previously published data revealed that 507 proteins were identified in both L3 larvae and adult *T. canis*, and 75 proteins were uniquely identified from the L3 larvae, including some C-type lectins (CTLs) (e.g., TES-32 and collectin-12) (Additional file 1: Table S1). It is hypothesized that *Toxocara* parasites possibly evade host immune response by secreting abundant functional CTLs [32]; among which, TES-32 is a major component of ESPs secreted by *T. canis* larvae [33, 34]. These findings suggested that the strategies adopted by *T. canis* for survival or development in the host may be different between its larval stage and adult stage. In addition, acetyl-coenzyme A synthetase and pyruvate dehydrogenase complex were identified in the larvae, whereas only pyruvate dehydrogenase complex was identified in adult *T. canis*, suggesting that *T. canis* may have stage-specific differences in energy utilization mechanisms. Further research on stage-specific proteins expression will enhance our understanding of the migration and development processes of *T. canis* in its hosts.

The protein kinases are a very extensive proteins family that share a conserved catalytic core common with both serine/threonine and tyrosine protein kinases, which play essential roles in a wide range of cellular processes, including division, differentiation, proliferation, apoptosis, and are responsible for phosphorylation [35]. Our previous genomic analysis predicted the presence of 458 protein kinases in *T. canis* genome [10]. In this study, we further identified 188 proteins with “protein kinase domain” at the protein level, including over 30 serine/threonine proteins. Moreover, GO annotation revealed that 253 proteins were enriched in “protein kinase activity” term under the “cellular component” category, and 258 proteins were enriched in “protein phosphorylation” term under the “biological process” category (Fig. 2b), suggesting that these protein kinases may play an important role in *T. canis*. Another significant protein group is the “major sperm protein (MSP) domain” proteins, unique to nematodes, which are essential for the amoeboid motility of nematode sperm, independent of actin [36]. In this study, we identified 67 proteins with “MSP domain” at the protein level, which may be the potential candidate proteins to control *T. canis* reproduction.

TFs are key regulators of gene expression, modulating the transcriptional activation or repression of target genes [37]. Our previous genomic study of *T. canis* revealed that *T. canis* encoded 355 TFs [10]. In this study, we identified 93 *T. canis* TFs belonging to 28 families. There are at least 42 zinc finger TFs in *T. canis*, including 27 ZBTB family TFs, 6 zf-C2H2 family TFs and 2 zf-GATA family TFs. By comparing our result with the previous study [12], we found that A0A0B2URD4 [3-methyl-2-oxobutanoate dehydrogenase (2-methylpropanoyl-transferring)], one of the CCAAT/enhancer binding protein (C/EBP) TFs family, can be identified from both L3 larvae and adult *T. canis*. By comparing with female adult *T. canis*, the expression level of A0A0B2URD4 was up-regulated 2.14-folds in male adult *T. canis*. The C/EBP TFs play crucial roles in regulating numerous significant biological processes, such as inflammation, cell proliferation and differentiation, energy metabolism, and signal transduction [38]. Further investigation into the biological functions of TFs, particularly those with stage- and gender-specific expressed TFs, will help to better reveal the development processes, spermatogenesis or egg generation of *T. canis*.

The genome data predicted that *T. canis* would produce at least 870 ESPs [10], and the proteome analyses identified 79 ESPs of *T. canis* L3 larvae [12, 29, 39]. In this study, we identified 385 “extracell protein”, seven of which correspond to previously identified ESPs from TES of L3 larvae, including A0A0B2V6Q8 (paramyosin), A0A0B2V815 (macrophage migration inhibitory factor-like protein, or l-dopachrome isomerase), A0A0B2W1X6 (transthyretin-like protein 46), A0A0B2UNP1 (laminin subunit beta-1), A0A0B2W1F7 (laminin-like protein lam-2), A0A0B2VHM0 (apolipophorin) and A0A0B2V9X0 (galectin). It is generally recognized that proteins consistently expressed throughout different stages of the life cycle may serve as effective vaccine candidate, although this idea is still speculative, given the relatively limited research in helminth immunology and vaccine development compared to other medical fields. Moreover, we found that none of these seven proteins identified from both L3 larvae TES and adult *T. canis* was PDAs in this study, suggesting that they are not gender-specific proteins and may play important roles in the process of *T. canis* migration and development, and these seven ESPs may be promising vaccine candidates for the prevention of toxocariasis [40–42]. Besides, some “cell membrane protein” (*n* = 429) are also important vaccine candidates of toxocariasis, such as A0A0B2VEA6 (enolase) and A0A183UM88 (cathepsin L), which need to be further investigated [43–45].

Compared to male adult *T. canis*, 682 up-regulated PDAs and 844 down-regulated PDAs were identified in female adult *T. canis*, including 139 proteins exclusively expressed in female *T. canis* and 272 proteins exclusively expressed in male *T. canis* in this study. It should be noted that during the mating process of female and male adult *T. canis*, the sperms of the male adult enter the body of the female, leading to the detection of male-specific expressed proteins within the female adult, such as some “major sperm protein (MSP) domain” proteins. MSPs constitute 15–20% of the total protein in nematode sperm and are absent from all other cell types in the worm [46]. Although the abundance of these male-specific expressed proteins is generally low in female adults and does not affect the type and quantity of PDAs, it will affect the statistics and analysis of gender-specific expressed proteins. This phenomenon will result in a lower count of male-specific expressed proteins than the actual situation. Previous study has shown a higher number of male-specific genes compared to female-specific genes, with 321 genes exclusively transcribed in female *T. canis* and 1467 genes exclusively transcribed in male *T. canis* [11]. Vaccination is still the most effective strategy for combating infectious diseases; however, the development of vaccines against multicellular helminths is riddled with obstacles due to its huge size, and constantly migration through various tissues under multiple life cycle stages [47]. To date, there are still no any reliable vaccine against zoonotic soil-transmitted helminths (STHs) [48]. Analysis and research of the PDAs between female and male adult *T. canis*, especially those gender-specific proteins related to spermiogenesis and egg generation, may facilitate the discovery of new vaccine candidates, such as major sperm proteins, chondroitin proteoglycan 1 and chitin-binding type-2 domain-containing protein. Many up-regulated PDAs, such as chondroitin proteoglycan 1, chondroitin proteoglycan 2 and chitin-binding type-2 domain-containing protein with “chitin binding domain”, were enriched in the GO terms of “aminoglycan metabolic process”, “chitin metabolic process” and “chitin binding”, indicating that they could contribute to the formation of the *T. canis* eggs in female adult *T. canis*.

Our previous study of the *T. canis* genome predicted that *T. canis* would produce 408 phosphatases [10]. In this study, we have also identified many phosphatases at the protein level. The protein tyrosine phosphorylation (PTP) is a prevalent post-translational modification that influences protein stability and regulates enzyme activity. Thus, maintaining an appropriate level of PTP is crucial for various cellular functions, including cell growth, proliferation, differentiation, and transformation, with particular importance during sperm capacitation [49, 50]. The PTP stands out as a critical intracellular signaling event that regulates sperm function, and the capacitated sperms show high levels of PTP, so sperm PTP is a meaningful indicator of capacitation [49, 51]. In the present study, many down-regulated PDAs, such as protein-tyrosine phosphatase, receptor-type tyrosine-protein phosphatase S and tyrosine-protein phosphatase non-receptor type 14 with “Protein-tyrosine phosphatase, receptor/non-receptor type” domain, were enriched in the GO terms of “protein tyrosine phosphatase activity”, suggesting that these proteins could play important roles in spermatogenesis and capacitation of *T. canis*.

In this study, we identified 21 TFs with differential abundance, belonging to 10 families, with 10 up-regulated PDAs and 11 down-regulated PDAs between female and male adult *T. canis*, as well as 3 TFs (e.g. A0A0B2VQ53, A0A183UMK5 and A0A183V3J6) exclusively expressed in female *T. canis* and 3 TFs (e.g. A0A0B2V842, A0A183VAA5 and A0A183V625) exclusively expressed in male *T. canis*. The roles of differentially expressed TFs between female and male adult *T. canis* may be critically important, particularly those that are sex-specific. In female *T. canis*, these TFs may potentially regulate genes involved in ovarian development and oocyte maturation, while in males, they may regulate genes associated with testicular development and spermatogenesis. These differentially expressed TFs may shape the biological characteristics of sexual dimorphism by regulating sex-specific gene expression, influencing reproductive system development, and modulating behavior and adaptability. However, research on the 21 differentially expressed TFs identified in this study remains limited to date. *T. canis* of different genders exhibit distinct physiological states and therefore may have different ESPs. In this study, we predicted the “extracell protein” by subcellular localization to explore the potential role of these PDAs. A total of 69 “extracell protein” were identified, including 46 up-regulated PDAs and 23 down-regulated PDAs, as well as 6 proteins specifically expressed in female adult *T. canis* (e.g. cysteine-rich motor neuron 1 protein) and 12 proteins specifically expressed in male adult *T. canis* (e.g. ephrin type-A receptor 3, ancylostoma secreted protein, orcokinin peptides type B and EGF_CA domain-containing protein). Further investigation of these proteins will enhance our understanding of the biological function of *T. canis*.

Helminths exhibit a sophisticated life cycle that includes various stages within the same host, with distinct antigen expression as they migrate and develop. They have developed mechanisms to modulate the host's immune responses, enabling them to escape immunological attack. By evaluating the efficacy of complex mixtures of parasitic lysates and secretions, and the individual proteins against STHs infection, researchers are becoming aware that there are still many difficulties and challenges in the vaccine development of STHs [48], for example, the lack of effective targets, high-quality genome data and comprehensive protein annotation information. Although 8565 *T. canis* proteins were identified based on *T. canis* protein database downloaded from UniProtKB (released on March 6, 2024) in this study, it should be emphasized that the information of a few proteins is still constantly being updated and changed in UniProtKB database, such as A0A0B2V815. In a previous study, A0A0B2V815 is called macrophage migration inhibitory factor-like protein based on the UniProtKB database [12]; however, it is called L-dopachrome isomerase in the current UniProtKB database (updated on January 24, 2024). As one of the major STHs and zoonotic parasite threatening animal and human health worldwide, the fundamental studies on molecular biology and pathogenesis of *T. canis* still need to be further strengthened to break through the bottlenecks in diagnostic methods and vaccine development.

Despite the comprehensive proteomic analysis conducted in this study, some limitations should be acknowledged. First, while the use of the Orbitrap Astral platform allowed for the identification of a substantial number of proteins in *T. canis*, the complexity and diversity of the proteome suggest that it is unlikely that the entire spectrum of expressed proteins was captured, particularly those with low abundance or transient expression. Second, while differential protein expression was identified between male and female adult *T. canis*, the functions of these proteins, especially TFs and ESPs, warrant further experimental validation.

## Conclusions

A total of 8565 somatic proteins were identified from female and male adult *T. canis* by quantitative proteomic approach based on the Orbitrap Astral platform, strengthening our knowledge on this enigmatic parasite. Clearly, some PDAs in female *T. canis* are involved in the formation of the *T. canis* eggs, such as chondroitin proteoglycan 1, chondroitin proteoglycan 2 and chitin-binding type-2 domain-containing protein. Whereas, some PDAs in male *T. canis* are related to the spermatogenesis and capacitation, such as protein-tyrosine phosphatase, receptor-type tyrosine-protein phosphatase S and tyrosine-protein phosphatase non-receptor type 14. The GO annotation analysis showed that many PDAs are involved in aminoglycan metabolic process, extracellular region, and protein tyrosine phosphatase activity. The KEGG pathway enrichment analysis showed that many PDAs are associated with adipocytokine signaling pathway, proximal tubule bicarbonate reclamation, and PPAR signaling pathway. These results provide critical insights into the pathobiology of *T. canis* and offer valuable resources for the development of novel intervention strategies for toxocariasis and related helminthiasis, especially from the perspective of sexual development and reproduction.

## Supplementary information

**Additional file 1: Table S1:** The overview of all identified adult *Toxocara canis* proteins. **Table S2:** The GO annotation analysis of all identified adult *Toxocara canis* proteins. **Table S3:** The KEGG enrichment analysis of all identified adult *Toxocara canis* proteins at the level 2 signaling pathways. **Table S4:** The domain analysis of all identified adult *Toxocara canis* proteins. **Table S5:** The transcription factors analysis of all identified adult *Toxocara canis* proteins. **Table S6:** The subcellular location analysis of all identified adult *Toxocara canis* proteins. **Table S7:** The proteins with differential abundance between the female and male adult *Toxocara canis*. **Table S8:** The GO annotation analysis of proteins with differential abundance between female and male adult *Toxocara canis*. **Table S9:** The KEGG enrichment analysis of proteins with differential abundance between female and male adult *Toxocara canis*. **Table S10:** The domain analysis of proteins with differential abundance between female and male adult *Toxocara canis*. **Table S11:** The transcription factors analysis of proteins with differential abundance between female and male adult *Toxocara canis*. **Table S12:** The subcellular location analysis of proteins with differential abundance between female and male adult *Toxocara canis*.

**Additional file 2: Fig. S1:** The coefficient of variancecumulative curve among the three biological replicates in each gender group.

**Additional file 3: Fig. S2:** The Venn diagrams showing the common and exclusive proteins between the two gender groups.

## Data Availability

The mass spectrometry proteomics data have been deposited to the ProteomeXchange Consortium (https://proteomecentral.proteomexchange.org) via the iProX partner repository [52] with the dataset identifier PXD051380.

## Abbreviations

Astral: Asymmetric track lossless
PDA: Protein with differential abundance
NGS: Next generation sequencing
LC–MS/MS: Liquid chromatography–mass spectrometry/mass spectrometry
DIA: Data-independent acquisition
DDA: Data-dependent acquisition
PCA: Principal component analysis
PRM: Parallel reaction monitoring
MSP: Major sperm protein
ESP: Excretory-secretory protein
CTL: C-type lectin
STH: Soil-transmitted helminth
PTP: Protein tyrosine phosphorylation
