# Supplementary figures and images for: Proteomics analysis reveals the differential protein expression of female and male adult Toxocara canis using Orbitrap Astral analyzer
Source: Infect Dis Poverty. 2024 Oct 9;13:73. doi: 10.1186/s40249-024-01246-9 (PMC11462720; doi:10.1186/s40249-024-01246-9)

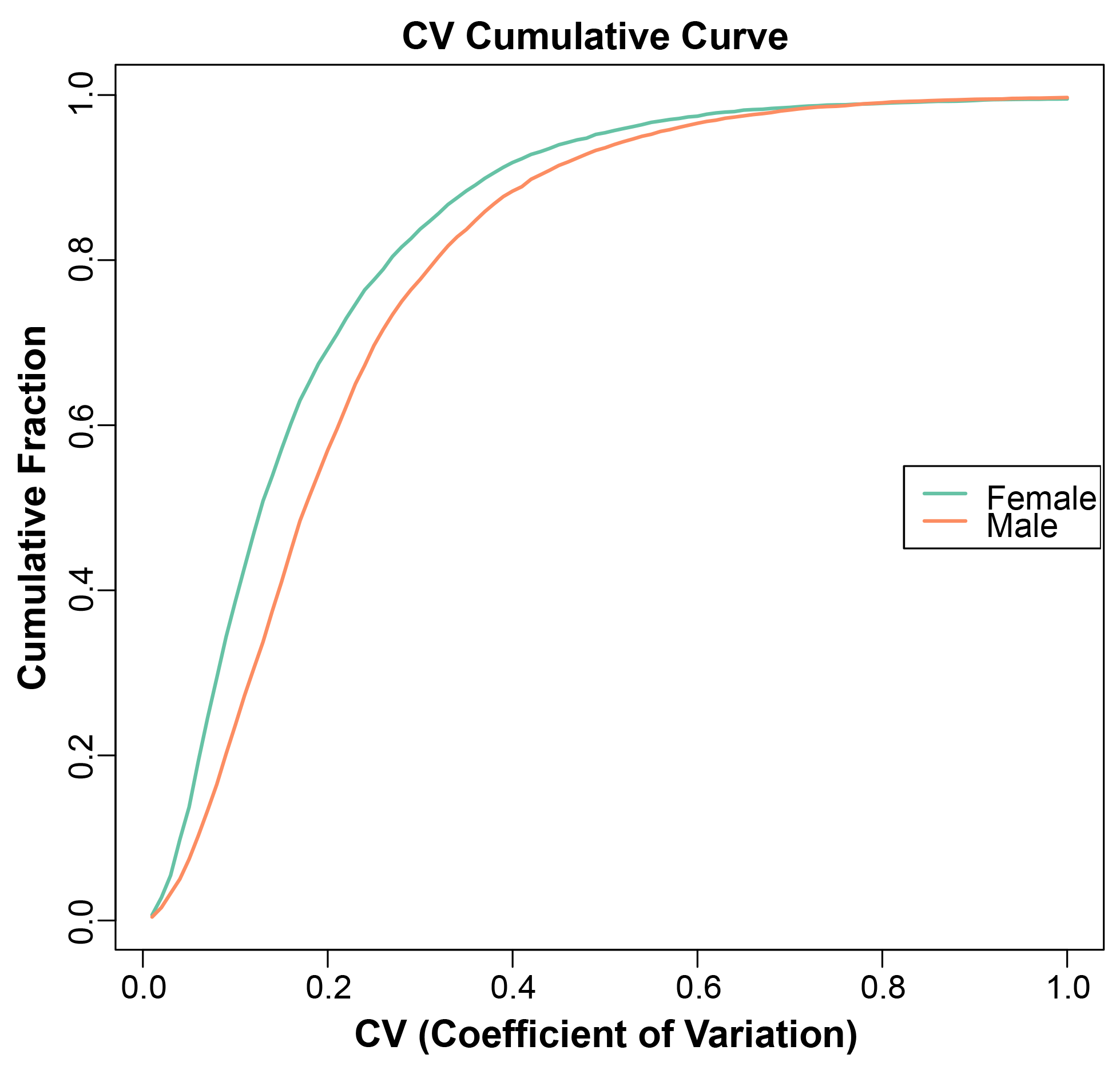

Supplement: Supplementary file 2 — Additional file 2: Fig. S1: The coefficient of variancecumulative curve among the three biological replicates in each gender group. [file 40249_2024_1246_MOESM2_ESM.tif]

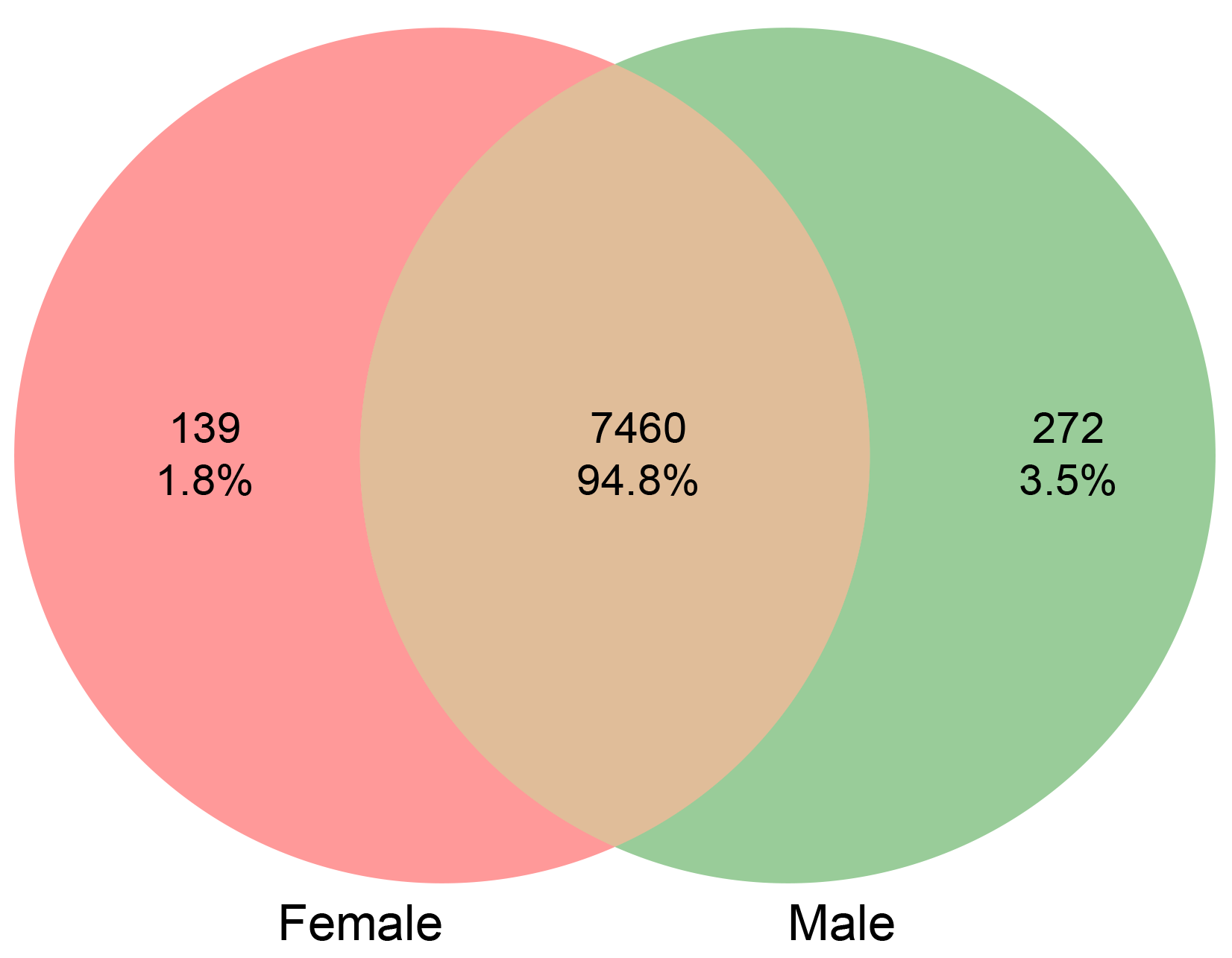

Supplement: Supplementary file 3 — Additional file 3: Fig. S2: The Venn diagrams showing the common and exclusive proteins between the two gender groups. [file 40249_2024_1246_MOESM3_ESM.tif]
